# A Systematic Review on Biomass Treatment Using Microwave-Assisted Pyrolysis under PRISMA Guidelines

**DOI:** 10.3390/molecules28145551

**Published:** 2023-07-20

**Authors:** Neyha Rubab Syed, Bo Zhang, Stephen Mwenya, Awsan Shujaa Aldeen

**Affiliations:** School of Energy & Environment, Southeast University, No.2 Sipailou, Xuanwu District, Nanjing 210096, China; 223227104@seu.edu.cn (N.R.S.); mwenya55674@seu.edu.cn (S.M.); aldeen8745@seu.edu.cn (A.S.A.)

**Keywords:** microwave, biomass, heating methods, pyrolysis, microwave-assisted pyrolysis, systematic review

## Abstract

Biomass as a renewable energy resource is a major topic on a global scale. Several types of biomass heat treatment methods have been introduced to obtain useful byproducts via pyrolysis. Microwaves are a practical replacement for conventional stoves and ovens to perform pyrolysis of biomass. Their rapid heating rate and user-friendliness make them a good choice for the pyrolysis process over conventional methods. The current study reviewed research articles that used microwaves for the pyrolysis process on different types of biomass. This study primarily provides comprehensive details about the pyrolysis process, especially microwave-assisted pyrolysis (MAP) and its feasibility for treating biomass. A systematic literature review, according to the PRISMA guidelines, was performed to find research articles on biomass treatment using MAP technology. We analyzed various research studies (*n* = 32), retrieved from different databases, that used MAP for pyrolysis on various types of biomass, and we achieved good results. The main goal of this study was to examine the usefulness of the MAP technique, comparing its effects on distinguished types of biomass. We found MAP’s effective parameters, namely, temperature, concentration of microwave absorber, moisture percentage of starting material and flow rate, microwave power and residence time of the initial sweep gas that control the pyrolysis process, and effect quality of byproducts. The catalytic agent in MAP pyrolysis was found to be useful for treating biomass, and that it has great potential to increase (nearly double) the production yield. Although MAP could not be used for all types of materials due to some challenges, it produced good results compared to conventional heating (pyrolysis) methods. We concluded that MAP is an effective method for reducing pyrolysis reaction time and improving the quality of value-added products. Also, MAP eliminates the shredding requirement for biomass and improves heating quality. Therefore, it is a viable method for reducing pyrolysis processing costs and should be applied on a larger scale than lab scale for commercialization.

## 1. Introduction

Fossil fuels are consumed in increasingly large quantities as the global energy demand and population rise, which worsens the energy dilemma and leads to more significant environmental concerns. As a result, residents are paying attention to the deployment of renewable resources and are showing interest in novel environmentally friendly options. Biomass is a renewable energy source that has benefits over conventional fossil fuels owing to its environmental friendliness and simple accessibility [1]. Biomass is defined as material or residue retrieved from living organisms. The term is used in various fields according to the situation, e.g., living organisms (L.Os) in ecology; matter from L.Os in bioenergy and later biomass are considered residuals either from plants, combination of plants and algae, or plants/animals. Mostly, the term “biomass” is used for plant residuals to represent bioenergy that contributes to the mitigation of climate change, e.g., gases generated from the burning of plant wood or residuals [2]. Nowadays, the term “biomass” is used to define a waste residual collected from industrial, residential, and agricultural areas. Raw materials and effluent left behind from industrial processing, solid waste from households, and residuals left behind after harvesting crops or dry plants leaves, etc., are considered biomass waste. The combined effect of biomass waste processing generated from the aforesaid sources leads to the creation of significant issues related to human health and sustainable environmental conditions [3,4,5]. There are three methods for converting the energy contained in biomass into biopower: combustion, bacterial decomposition, and transformation into vapor or liquid fuel. The most common method for converting biomass into useful energy is direct combustion [6]. The thermochemical conversion of biomass comprises both pyrolysis and gasification [7,8]. Each method has its own limitations and advantages. Biomass could yield higher-value end-products by using thermochemical conversion processes such as microwave-assisted pyrolysis. The amount and quality of the world’s energy consumption are affected by a number of variables, including rising populations and expanding economies. The global population has been growing at a staggering pace [9,10,11,12,13], and the utilization of energy sources has significantly increased in recent decades [14,15]. The research community has expressed that the primary cause of global warming is the use of fossil fuels. Increasing carbon dioxide (CO_2_) emissions can be attributed to both the increasing carbon intensity of energy supply and increasing energy intensity of economic activity [16,17]. In light of the growing demand for energy as a result of an expanding economy and population, researchers, industrialists, and governments have been investigating alternative, less expensive energy sources to mitigate the threat of climate change and global warming [18,19,20].

The method used to treat biomass is of critical importance due to the indirect effect on human wellbeing by affecting the environmental ecosystem. An incinerator is used in the thermal treatment of biomass to perform the incineration process. Mostly, the industrial sector uses incinerators for the burning of biomass, which effects the atmosphere and has become a source of air pollution. Also, the high cost of incinerators, their maintenance, and the handling of residual leftovers after biomass burning creates difficulties in the use of incinerators. Therefore, an alternative method for treating biomass that also reduces the amount of residual leftovers after treatment is direly needed [21,22,23]. The process of incineration induces chemical changes that may produce harmful products that can escape through the stack, causing air pollution, or that can remain in the bottom ash, eventually finding its way into landfills. In developing countries, there are still concerns from experts and local communities about the environmental effect of incinerators. Currently, waste incinerators dispose of ash by sending it to landfills, which is both costly and takes up physical space. In some developed countries (USA, UK, Europe, China, Japan), incinerators built just a few decades ago often did not include a material separation process for removing hazardous, bulky, or recyclable materials before combustion. These facilities tended to risk the health of plant workers and the local environment due to inadequate levels of gas cleaning and combustion process control. Additionally, nearby residents often express displeasure with incinerators’ effects on their quality of life, including increased traffic, odors, and noise. People frequently have to cover their windows and stay inside throughout the summer since the odor becomes unbearable when the weather warms up [24]. To overcome these problems, the pyrolysis process was introduced and used to treat biomass. This process achieved its objectives by (1) reducing the volume of leftover residuals in the case of the incinerator and (2) generating useful byproducts. Also, the byproducts generated from the biomass pyrolysis process have a negligible effect on the atmosphere and even provide electricity and heating through the combustion of particulate emissions. Biomass pyrolysis, which occurs in an oxygen-depleted environment, uses heat to convert biomass feedstock into bio-oil, charcoal, and syngas [25]. The organic matter is thermally destroyed by breaking down chemical linkage bonds in an inert atmosphere [26]. It is an environment in which powder bed fusion may occur without the presence of reactive gases like oxygen and CO_2_, which are naturally present in the air but might potentially contaminate the reaction [25]. The final products of the pyrolysis process are useful products in gaseous form (synthesis gas—a mixture of hydrogen and carbon monoxide), liquid form (bio-oil), and solid form (bio-char), which increase system efficiency and decrease the effect of emissions on the environment.

An electric furnace was used for pyrolysis, and nitrogen (N_2_) was used as a cleaning agent. Various instruments are used to perform the pyrolysis process. Because of the advantages of microwave heating over more traditional heating methods, extensive research on microwave-assisted pyrolysis of biomass waste has been conducted over the last decade [26,27,28,29,30]. Various microwave systems may now be constructed, developed, and optimized to treat biomass into marketable end-products [31,32,33,34]. Many research studies have been conducted in recent years to investigate whether microwaves can be used to treat biomass during the pyrolysis process and transform biomass into useful products such as bio-fuel (liquids) and bio-char (solids) [35,36,37,38]. The following studies (oil palm shell [39], paper deinking residue [40], plastics [41], wood sawdust [42], oily sludge [43], sewage sludge [44], cellulose [45], gumwood [46], and rice straw [47]) are recently reported in literature which have used MAP technology to convert biomass into useable products.

Using microwave-assisted pyrolysis (MAP) to treat biomass has several obstacles that should be solved. Primarily, there are two main issues with microwave biomass pyrolysis processing: one is the microwaves themselves, and the other is the processing of biomass materials [48,49,50]. Biomass from different sources can be converted using various methods, but microwaves outperform traditional methods for treatment, pyrolysis, and heating processes [51,52,53,54,55,56,57,58,59,60,61,62,63,64,65,66,67,68,69,70,71]. The ability of a material to convert into energy depends on its dielectric properties. Dielectric heating is a volumetric process because heat is generated inside the material by the selective absorption of electromagnetic energy. However, microwave pyrolysis cannot be used for all materials, e.g., transparent and dry materials, but it works efficiently in materials with a high moisture content. In addition, refined materials along with absorbing agents in the microwave assist in increasing the overall temperature of the reaction. The difficulty of monitoring the temperature in a microwave environment and the thermal damage to treated materials due to non-uniform heating behavior are challenging issues. Because the homogeneity of the microwave field may be improved by enlarging the cavity, a specific instrument design is required. Additionally, microwave leakage poses a risk to human health, necessitating safety measures and cautious handling. Therefore, the objectives of this research are (1) to study the MAP process for biomass treatment and (2) to examine factors that affect the quality of the final product using the MAP process.

## 2. Pyrolysis Process

Pyrolysis is a thermochemical process that can be used to treat any organic (carbon-based) product. It can be performed on both pure and mixture of materials. During the treatment process, the material is subjected to high temperatures and separated into distinct molecules through chemical and physical processes in the absence of oxygen [72]. Due to the poor thermal stability of chemical bonds in materials, breakdown occurs, and heat can dissolve them. New molecules are formed as a result of thermal breakdown. This allows products to be obtained that have a distinct, and often superior, quality to the original residue. Because of this property, pyrolysis is becoming great choice in today’s business and enabling considerably higher value to be extracted from ordinary materials and waste [73,74]. Thermal treatment is commonly related to pyrolysis. However, unlike combustion and gasification processes, which entail complete or partial oxidation of the material, pyrolysis is based on heating in the absence of air. This results in an endothermic process with significant energy content obtained in the products. Pyrolysis always results in solid (charcoal, bio-char), liquid (bio-oil), and non-condensable gases (hydrogen (H_2_), carbon monoxide (CO), and CO_2_). Because the liquid phase of pyrolysis gas is only removed after cooling, these two streams can be used together in certain applications when feeding hot syngas straight to the burner or oxidation chamber [75,76,77]. Pyrolysis can be classified as either slow, fast, or flash [78] based on the following variables: RT = residence time; HR = heating rate; Te = temperature; VR = vapor resentence time. Table 1 provides a categorization of the pyrolysis process using the numeric range of these variables. 

Fast pyrolysis is preferable since it produces a higher percentage of liquid waste [79,80]. Numerous fast pyrolysis technologies have focused their commercial-scale applications on rapid catalytic pyrolysis, which has encouraged integrated process development initiatives [81,82]. Most volatiles develop in the biomass pyrolysis between 250 and 500 °C [83], giving pyrolysis a broad temperature range (400 to 1200 °C). Biomass pyrolysis at 400 °C recovers just a small amount of volatiles as bio-oil and syngas but produces a lot of bio-char. The final products of the biomass pyrolysis process are syngas, bio-oil, and bio-char. A slow pyrolysis temperature prevents the thermal cracking and water shift processes. There is a linear rise in H_2_ content between 500 and 900 °C, a more gradual increase in CO and H_2_ contents between 700 and 900 °C, and a minor drop in H_2_ contents between 800 and 900 °C. Because of these alterations, the value of gas products has increased. Thormann and de Oro [84] stated that when fast pyrolysis is performed in an optimal environment, high yields of liquid products may be effectively achieved.

**Figure 1:**
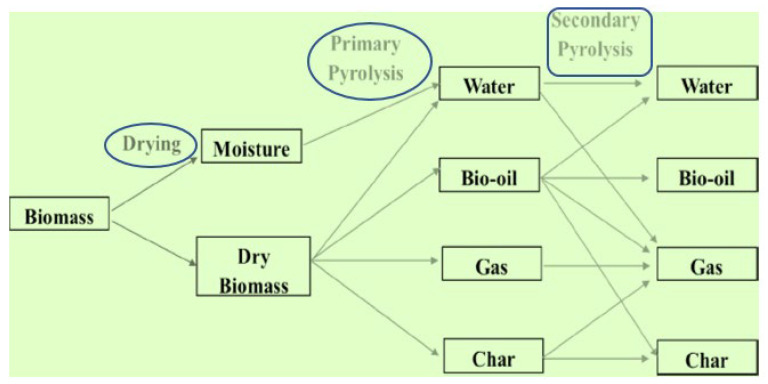
General formation of biomass into gaseous, liquid, and solid state.

The mathematical formulation of biomass conversion can be found in previously published research articles [85,86,87]. However, the general formation of biomass into gaseous, liquid, and solid forms is shown in Figure 1. The graphic below clearly illustrates the main and secondary reactions. As shown in Figure 1, dry biomass can be converted into various products such as bio-char, biogas, bio-oil, and oily water during the primary reactions, whereas during the secondary reactions, the same products obtained during the primary reactions are converted into similar products; however, this time, the bio-char is converted into gaseous products as well as more refined bio-char. The bio-oil is changed into oily water, additional gaseous products are formed, bio-char is formed, and certain sections remain the same as the bio-oil. As the reaction progresses, the oily water is transformed into additional gaseous products, while the remaining water stays as oily water.

### Biomass Pretreatment for Pyrolysis

**Figure 2:**
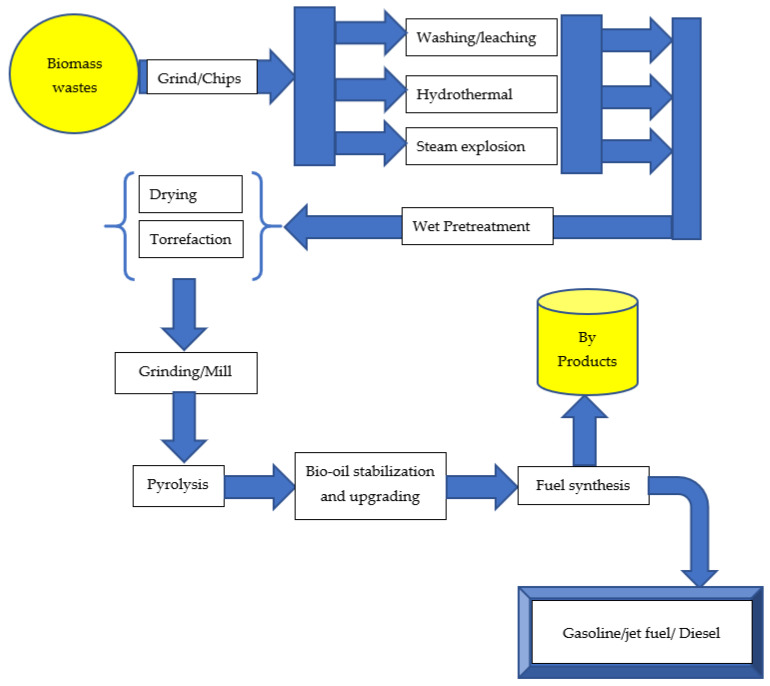
Biomass pretreatment converts biomass into useful components.

Biomass pretreatment can be performed before pyrolysis to improve the process’s selectivity for desired products. Cellulose, hemicellulose, and lignin are the three primary components of lignocellulosic biomass. Pretreatment modifies the lignocellulosic biomass material’s internal structure by decrystallizing the cellulose and removing the lignin and hemicellulose [4,28,29] to improve the efficiency of the subsequent biomass conversion. Biomass may be thermally broken down into liquid, solid, and gaseous phases. The conditions of biomass thermal decomposition have set limits on the yield of the three-phase products. Bio-oil produced through the pyrolysis process often represents the largest amount obtained compared to other processes. Higher heating rates may increase bio-oil yields; nevertheless, the increased oxygen and water content of bio-oil makes it less desirable for direct use as transportation fuel. The physicochemical characteristics of the biomass and the pyrolysis parameters (temperature, heating rate, particle size, residence duration) determine the bio-oil’s quality and yield. Biomass has a variety of physicochemical characteristics, including a high heating value, grindability, flowability, high volatile content, and low ash content. Wet and dry pretreatment techniques have the potential to enhance the physicochemical qualities of biomass. Wet pretreatment processes include washing and leaching, the steam explosion, and the hydrothermal method, whereas dry pretreatment processes include drying and torrefaction. These pretreatment methods may enhance the physicochemical qualities of biomass, resulting in a higher-quality and more abundant bio-oil after pyrolysis. The conversion of biomass into useful products via the pretreatment process is shown in Figure 2. These products can be used in a variety of sectors, including energy generation, chemical production, food production, and medicinal components. Additionally, physical, chemical, and biological pretreatment techniques could be used solely or in combined form to pretreat biomass [88]. Zadeh and colleagues [1] provided a comparative analysis on the pretreatment techniques and their impact on biomass pyrolysis.

## 3. Microwave-Assisted Pyrolysis

**Figure 3:**
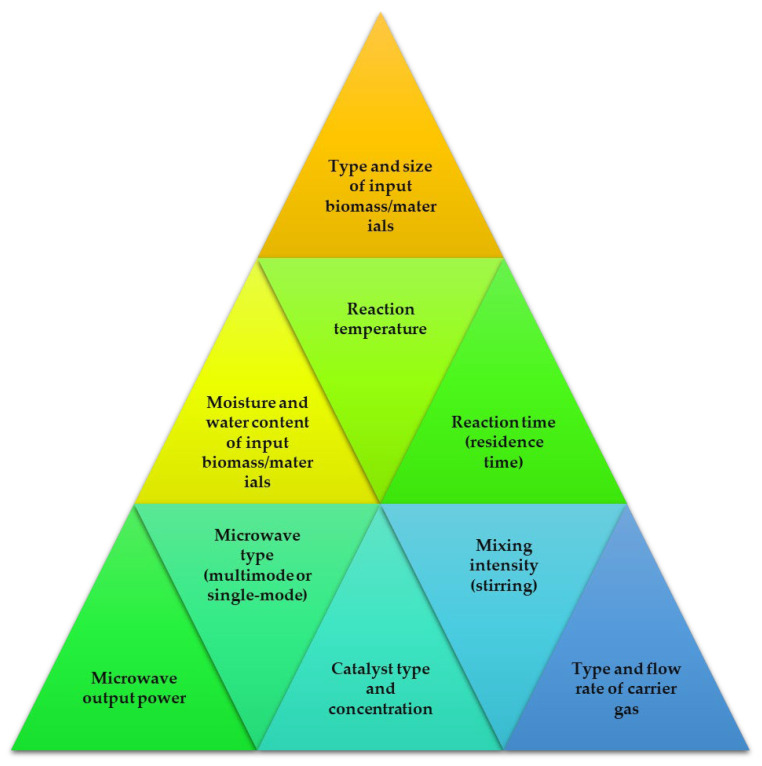
Factors contributing to the microwave-assisted pyrolysis reaction processes.

When it comes to improving and speeding up chemical processes, the microwave method is great choice. Due to excellent heat transfer patterns, the reactions can be performed in less time compared to conventional methods [89]. It has quickly become one of the most promising new technologies because of the time and energy saved by shortening the residence time and speeding up the chemical processes. Electromagnetic radiation, known as microwaves, has wavelengths between one millimeter and one meter. The microwave portion of the electromagnetic spectrum spans from 300 MHz to 30 GHz, lying between the infrared and radio frequency portions of the spectrum [90]. To avoid interfering with RADAR signals and telecommunications wavelength bands, most microwave systems run at 2.45 GHz or 900 MHz [91]. Several crucial elements in the MAP process have an impact on the final product’s yield and quality. Optimal results in terms of quality and conversion rate may be achieved by adjusting these factors, as shown in Figure 3. 

Energy and money savings may be possible using MAP technology. It has been shown to be an effective method of recycling unwanted materials and creating useful products from biomass and bio-waste. Despite the method’s promise for future industrialization, it has not yet been implemented at a commercial scale. The benefits of MAP technology as a viable alternative to traditional methods (incinerators) are outlined below.

The primary benefits of this technology are its speed, selectivity, and uniformity in heating, which facilitate the treatment and usage of inhomogeneous wastes and biomass of significant size. Forest and agricultural wastes and residues, municipal solid waste (MSW), and MSW sludge are all examples of high-moisture biomass which can be treated easily with MAP technology. The effectiveness of the product, the chemical processes, and the process as a whole improved using MAP. On other hand, the adaptability of process, and the mobility of the necessary equipment made easy to use MAP technology for biomass treatment.

Gro Harlem Brundtland presided over the United Nations’ World Commission on Environment and Development, which was established to determine solutions for human activities. This research led to the publication of a book titled “Our Common Future” [92], which serves as a roadmap for environmentally responsible growth [93]. In this book, sustainable development is explained as “meeting the requirements of the present generation without compromising the needs for future generations”. The worldwide demand for bio-products, including bio-oil, bio-char, and syngas, has increased as a result of the need to meet these objectives and criteria. The adaptation of MAP technology offers many developing countries a way out of poverty by creating new opportunities for research and development (R&D), generating income for farmers, reducing greenhouse gas emissions, increasing crop yields and land productivity, and fostering agricultural sustainability [94].

### Application and Limitation

**Figure 4:**
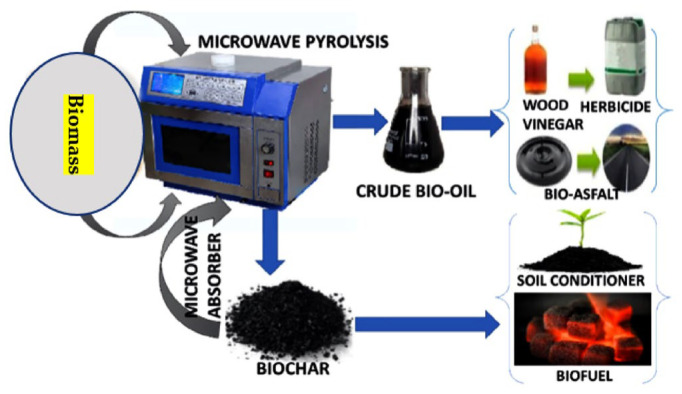
Microwave-assisted pyrolysis creating useful products out of biomass.

MAP for biomass treatment is a novel thermochemical conversion method. The oxygen content and heating value of the bio-oils produced are both greater than those produced by traditional biomass pyrolysis. Because dielectric characteristics influence microwave absorption, they play a significant role in the MAP process. By using exogenous microwave absorbents and reaction catalysts, it is possible, via MAP, to increase bio-oil output and quality via rapid heating, high pyrolysis temperatures, and catalytic breaking of big molecules. However, the bio-oil production may be reduced if the pyrolysis temperature is too high, since this will trigger secondary processes that convert the volatiles to gases that cannot be condensed. MAP technology has significant benefits over traditional pyrolysis techniques and, hence, a promising future. Microwave heating, in particular, is a well-established method that can be quickly and accurately applied. Cost-effectively producing high-quality bio-oils and commercializing the concept requires more work in the areas of catalyst screening and applications, co-pyrolysis of biomass with complimentary microwave heating properties, pilot research, and equipment development. The potential applications of the MAP process for creating useful products from biomass are shown in Figure 4. 

## 4. Systematic Literature Review

**Figure 5:**
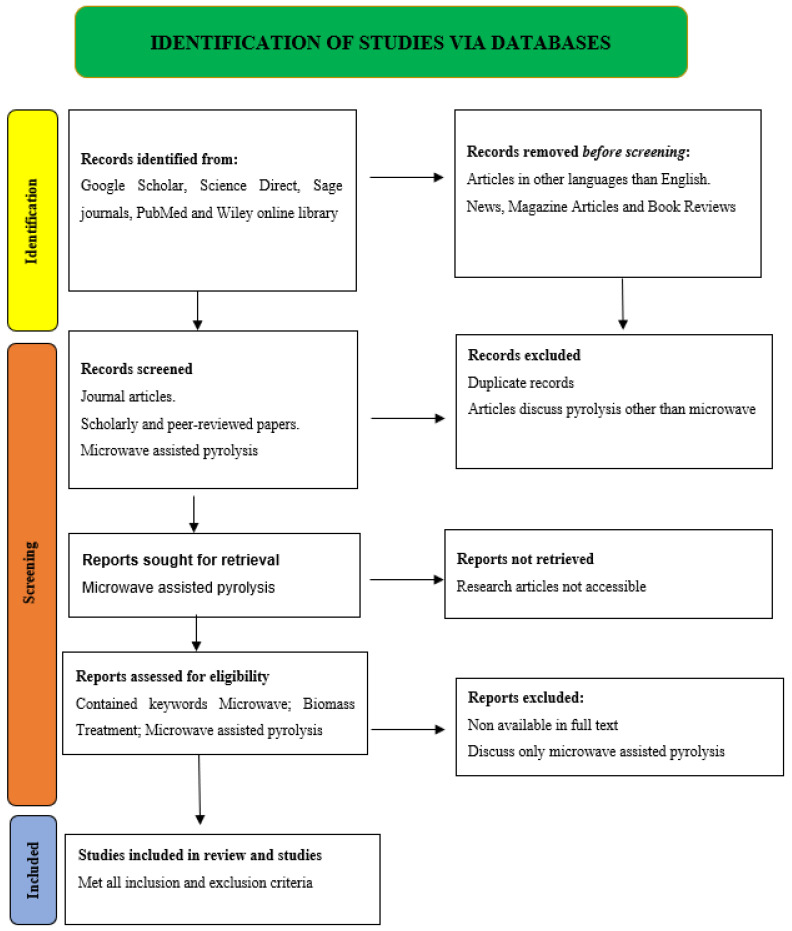
PRISMA diagram of current study.

This systematic review followed the PRISMA guidelines for systematic reviews and meta-analyses. The PRISMA schematic diagram is prepared according to Figure 5 standards. Considerations for the PRISMA flow diagram understanding included the following:

(1) Data sources: Google Scholar, Science Direct, Sage journals, PubMed, and Wiley online library databases are used with the following titles and keywords: microwave-assisted pyrolysis, biomass, pyrolysis processes, microwave processes, etc.
(2) Article screening: the chosen databases include duplicate, irrelevant, full-text unavailable, and non-English entries. These are deleted from identifiable records.
(3) Article inclusion: The investigation included papers on microwave-assisted pyrolysis, biomass, pyrolysis processes, and microwave processes.

As of 5 May 2023, the last Google Scholar, Science Direct, Sage journals, PubMed, and Wiley online library search for “microwave assisted pyrolysis” yielded 35,277 publications. Google Scholar returned 15,000 results, Sage Journals 128 results, Science Direct 14,838 results, PubMed 250 results, and Wiley Online Library 5061 results for research studies. The review excluded local language articles, hearings, and non-peer-reviewed publications. Finally, the 32 research articles relevant to MAP technology for the treatment of biomass included in this review examine its effectiveness and affirm that it produces good-quality products.

As previously stated, the MAP process is one of the most promising ways to boost and speed up chemical processes for biomass conversion. Due to a good heat transfer mechanism, the reactions may be performed in less time compared to more traditional heating processes [94]. Since it shortens the residence time and speeds up the chemical processes, both of which result in significant savings of energy as well as money. The following studies affirmed the use of MAP technology to convert different types of biomass into useful products.

Zhao et al. [95] examined the impact of temperature on microwave-induced pyrolysis of wheat straw as a biomass input. A domestic microwave with a frequency of 2.45 GHz and a power output of 3000 W was used. A thermocouple was installed to monitor the pyrolysis temperature in the microwave reactor. The effectiveness of MAP technology was found to be highly temperature dependent. The yield of gas products increased from 17.69 to 22.27 wt% when the temperature was increased from 400 °C to 600 °C. The average pore size decreased from 282.16 to 46.64 nm, while the specific surface area of the solid fraction rose from 0.89 to 9.81 m^2^/g, and the pore volume rose from 0.006 to 0.012 cm^3^/g. Hu et al. [96] examined the impact of microwave power level and catalysts, including activated carbon (C_ac_), CaO, and SiC, on the metabolites of *Chlorella vulgaris*. Guangzhou Wancheng Microwave Equipment Co., Ltd., Gaungzhou, China, utilized a microwave (2.45 GHz, 3750 W) in the experiment. The microwave oven’s carrier gas was 300 mL/min (mins) of N_2_ gas from a canister connected to the reactor’s three apertures. The maximum volume of the reactor was 500 mL. The gases escaped through the bottom neck of the container and were captured in flasks equipped with thermocouples (TCs). When microwave power was increased from 750 W to 2250 W and temperature increased from 200–800 °C, the gas product output increased, while the solid fraction production decreased. The results showed that a microwave exit power of 1500 W produced the highest bio-oil yields (35.83 wt%), while a microwave exit power of 2250 W produced the highest gas yields (52.37 wt%). At higher microwave power levels and higher concentrations of catalysts, C_ac_ was found to be the most efficient at diminishing solid residue and enhancing gas production. Using the optimal amount of C_ac_ (5%), maximized biofuel production (87.47%) and increased gas output (75.66%) could be achieved.

Ren et al. [97] examined how reaction time and temperature affect the production of syngas, bio-oil, and charcoal from Douglas fir sawdust particles with the help of a microwave. The synthesis of volatiles and charcoal was optimized using central composite design (CCD) and response surface analysis (RSA). In this research, a Sineo MAS-II bulk microwave oven with a maximum power output of 1000 watts was utilized. Each mixture of 400 g of pelletized Douglas fir sawdust was microwaved for one minute in a one-liter quartz reactor. The greatest concentrations of volatiles (bio-oil and syngas) were observed after 15 min at 471 °C when the reaction parameters (time and temperature) were altered. Depending on the pyrolysis parameters, volatiles and bio-oil yields varied between 39.3–68.8 wt% and 33.8–57.8 wt%, respectively. Depending on the reaction parameters, the yields of charcoal and syngas achieved were 31.2% to 60.7% and 7.9% to 15.0%, respectively. In the study of Chen et al. [98], NaOH, Na_2_CO_3_, Na_2_SiO_3_, NaCl, TiO_2_, HZSM-5, H_3_PO_4_, and Fe_2_(SO_4_)_3_ were employed as additives, and microwave irradiation at 470 °C with Silicon carbide (SiC) as an absorber was used to conduct microwave heating. For 15 min, a 0.2 m^3^/h N_2_ discharge was used to purge the reactor of air. All eight additives increased the solid fraction production, while the gaseous product yield decreased, and the liquid product yield remained relatively constant. These additives resulted in the production of H_2_, CH_4_, CO, and CO_2_ during pyrolysis, with sodium being the most efficient. NaCl, TiO_2_, and Fe_2_(SO_4_)_3_ were the only three reagents that did not reduce CO_2_ and CH_4_ while increasing H_2_ production. Except for HZSM-5 and Na_2_SiO_3_, all of the additions were successful in reducing CO levels. The most effective at increasing acetol production were the alkaline sodium compounds (NaOH, Na_2_CO_3_, and Na_2_SiO_3_). HZSM-5 had no effect, and TiO_2_ caused acetol production to decrease.

Omar et al. [99] investigated the use of microwave irradiation to characterize the empty fruit bundle (EFB) of oil palm for pyrolysis. In addition to demonstrating that a potassium concentration of 12.8% influenced microwave absorption positively, the dielectric properties and moisture content were observed to be proportional up to 60% at 2.45 GHz. Hussain et al. [100] used the microwave–copper interaction method to co-pyrolyze waste polystyrene and Makarwal coal at high temperatures. Co-pyrolysis yields better results than single pyrolysis, with a 6 percent gas component, 10 percent water (including sulfide), and 66 percent oil. In order to remove bio-oil from sewage sludge, Tian et al. [101] used MAP technology. Bio-oil was produced from wastewater by using the optimum microwave exit power and product mass balance. Between 400 and 600 watts of electricity was ideal for bio-oil production. Between 600 and 800 W of departing power, gas output rose, while bio-oil production decreased. Beyond 800 W, gas creation rose, but the solid fraction reduced, while bio-oil production remained unchanged. Bio-oil output was maximized at 49.8% when microwaves were used at 400 W for 6 min at 35.5 MJ/kg and 929 kg/m^3^. When the microwave departure power was raised from 200 W to 1200 W, the production of biogas rose from 4.4% to 60.21%, while the output of solid fraction dropped from 77.3% to 32.6%. Du et al. [102] used MAP technology on rice chaff and sawdust. The results of varying the exit power of the microwave oven (160–800 W), the heating duration in the microwave oven (0–25 min), and the concentration of the catalyst (from 0 to 0.5 catalyst to biomass) were studied. Two types of ionic liquids (IL-C and IL-B) were used as catalysts. At an exit microwave power of 640 W, bio-oil production from rice straw was 38%, and at an IL-C/straw ratio of 0.15, it was 42%. The bio-oil yield was 34% after 20 min in the microwave at a ratio of 34% IL-B/sawdust. IL-C and IL-B stimulated bio-oil synthesis in 2 and 8 min, respectively. The production of bio-oil from rice straw stabilized after 20 min of microwave irradiation (both catalysts).

Zeng et al. [103] studied the morphology and production of carbon nanostructures (nanospheres and CNTs) during catalyst-free microwave-assisted methane pyrolysis. The experiment made use of a 2.45 GHz home microwave oven with a vertical quartz tube. There was no need for additional minerals to encourage the production of nanostructures in the carbon/carbon composite microwave absorber (MA) material utilized in the pipe. Flushing N_2_ through the hole removed oxygen prior to testing; the quartz wall material underwent further testing after being exposed to a CH_4_/N_2_ (1:4) mixed gas flow reaction for 60 min. Some of the experimental conditions that impacted the resultant nanostructures were the methane-to-N_2_ ratio, the reaction temperature, and the overall gas flow rate. Huang et al. [104] used MAP to turn rice chaff into hydrogen-rich propellant gas. A 2-KW single-mode microwave was used to perform experimental analysis. N_2_ was used as the pyrolysis carrier gas, and the chamber was outfitted with a quartz reaction tube and a sample receptacle. Hydrogen (H_2_), carbon dioxide (CO_2_), carbon monoxide (CO), and methane (CH_4_) were the primary gases released, with an estimated 60% of the output usable as bioenergy. Fu et al. [105] used the MAP of methane to construct SiC nanowires on a quartz plate without using a catalyst. Tests were conducted using a 2.465 GHz microwave pyrolysis reactor with three 1 kW magnetrons in a laboratory environment. The effect of reaction gas concentration and temperature on the shape of SiC nanowires was investigated. The nanowires made using this cutting-edge technique were very pure. After sieving, four dry fractions and two wet fractions ranging in size from fine dust to 7–12 mm were achieved. Andersson et al. [106] analyzed six distinct fractions of discarded electrical and electronic equipment waste to generate bio-oil and syngas using MAP technology. The results exhibited that MAP is an effective method for transforming waste into valuable products. Chemat and Poux [107] investigated the pyrolysis of urea into cyanuric acid using MAP technology in a heterogeneous environment. The results indicated that graphite enhanced product yield from 15.2% to 61.2% at 300 °C, and from 4.6% to 9.6% at 200 °C. Heterogeneous pyrolysis was responsible for the faster response times. 

In order to investigate MAP effectiveness on oil palm shell and fiber (OPS, OPF) biomass, Salema and Ani [108] used char as an MA. The research was conducted using a modified household microwave oven set at 2.45 GHz and 1 kW. A steel distribution plate, a quartz glass reactor, and two k-type metallic TCs were used to monitor both the outside and inner temperatures of the furnace. At a constant microwave exit power of 450 W and a reaction time of 25 min, the impact of raising the OPS/OPF-to-MA ratio from 1:0.25 to 1:1 was investigated. Increasing the biomass-to-MA ratio from 1:0.25 to 1:0.5 is suitable for maximizing bio-oil and gas production in OPS and OPF while avoiding char formation. Changing the biomass-to-MA ratio from 1:0.5 to 1:1 boosts char production while lowering bio-oil output and maintaining gas yield. MAP technology on distiller’s dried grain with soluble (DDGS) was studied by Lei et al. for its potential to provide bio-char, bio-oil, and syngas [109]. In this investigation, the effects of reaction time, temperature, and microwave power were examined by means of response surface methodology (RSM). The recovery rates for bio-char, bio-oil, and syngas all varied between 22.6% and 62.2% depending on the pyrolysis conditions. Increasing the reaction temperature and time enhanced bio-oil and syngas yields while decreasing charcoal yields. Wan et al. [110] investigated the catalyst effects on the MAP of dry maize stover and aspen wood pellets. MAP was performed on a mixture including 100 g of dried biomass and 8 g of powders/crystals containing K_2_Cr_2_O_7_, Al_2_O_3_, KAc, H_3_BO_3_, Na_2_HPO_4_, MgCl_2_, AlCl_3_, CoCl_2_, and ZnCl_2_. Each test lasted for 20 min and was performed in a microwave reactor operating at 450–550 °C and 2450 MHz, with 875 W continuous input power. Charcoal and gas production were both reduced, while bio-oil production was increased by the addition of these substances (KAc, Al_2_O_4_, MgCl_2_, H_3_BO_3_, and NaHPO_3_). When absorbed by a catalyst, microwaves may accelerate the heating process. The MAP of dried maize stover (4 g/100 g sample) and aspen wood pellets (8 g/100 g sample) resulted in bio-oil yields of 42.70 to 71.11% and 41.12 to 71.4%, respectively; catalysts Na_2_HPO_4_, K_2_Cr_2_O_7_, and Al_2_O_3_ generated the highest yields of bio-oil (44%), syngas (37%), and charcoal (30%). Bio-oil generation from biomass (Chlorella sp.) was studied by Du et al. [111] utilizing MAP technology. All of the pyrolysis took place in a microwave oven (Panasonic, NN-SD787S) set to 2.45 GHz at an exit power of 1.25 kW. For these analyses, a 500 mL quartz flask was filled with 500 mL/min of N_2_. Biomass pyrolysis char, with an effective ratio of 1:5 (char to biomass), was utilized as the MA to improve heating quality. The production of bio-oil was 28.6% at an exit microwave power of 750 W. Adding more fuel reduced performance. The gas production was enhanced by 12% when the power was raised from 500 to 1250 W.

For the pyrolysis of used motor oil, Lam et al. [36] used MAP technology. An oil waste reactor made of carbon particles and quartz, heated in a microwave oven at 5 kW, was utilized. The pyrolysis temperatures ranged from 250 °C to 700 °C, and the carrier gas was N_2_ at a flow rate of 0.1 to 0.75 L/min, with a heating rate of 60 °C/min. The temperature of the reaction was monitored by two TCs placed at the top of the carbon bed and the bottom of the stainless-steel stirrer shaft. At 250 mL/min N_2_ and 5.0 kg/h waste oil input rates, 88 wt% pyrolysis oil was produced. When raising the waste oil feed rate from 0.4 kg/h to 5.0 kg/h and maintaining a N_2_ purge rate of 250 mL/min, the yield was maximized. MAP with C_ac_ was used by Bu et al. [112] to extract phenol and phenolics from Douglas fir (lignocellulosic bio-mass). The reaction temperature, duration, and C_ac_-to-biomass ratio varied from 1.32 to 4.86, and the microwave input power was 700 W with a heating rate of 60 kelvin (K)/min. Phenol (38.9%) and phenolic (66.9%) concentrations were both high while using a catalytic ratio of 3:1, a reaction temperature of 589 K, and a retention time of 8 min. The MAP of automobile engine oil created incondensable gases, which were examined by Lam et al. [113]. The identical apparatus was utilized for the experiment as in Ref. [36], but a membrane filter was installed to clean the pyrolyzed volatiles of any metallic solid residue before they entered the condensation apparatus. The by-product gases included H_2_ and H_2_CO concentrations of 19–35 vol%.

MAP was used by Quan et al. [114] to investigate high-phenol biofuels. In this investigation, phenolic-rich pyrolysis oil was generated using the microwave pyrolysis system described in Ref. [112]. The effects of time, temperature, and catalyst-to-feedstock ratio were investigated. Because C_ac_ decomposes lignin, MAP has promise for the bioconversion of Douglas fir (biomass feed-stock). Phenol (at 38.9%) and phenolics (at 66.9%) were found in quite high proportions in the biofuels. Volatile yields were significantly boosted by using C_ac_. The gas output was reduced, while the liquid yield increased from 32.3% (315.85 °C) to 38.8% (399.85 °C). Lam et al. [115] examined the MAP of vintage vehicle engines. The reaction temperature was increased from 400 to 700 °C, while a quartz reactor containing 1 kg of carbon was heated in a microwave oven with a carrier gas flow rate of 0.2 L/mins of N_2_. The most lucrative products were obtained at 600 °C. The yield for liquids was 75% at this temperature, whereas the yields for gas and solids were the lowest. The metal pollutants in the recovered liquid oils were drastically cut down on, with Cd reductions of 46%, Cr reductions of 32%, and Cu, Ni, Pb, Zn, and Fe reductions of 93% to 97%. Bio-oil was extracted from sewage sludge by Lin et al. [116] using MAP technology. The quality and quantity of bio-oil were investigated, along with the effects of different reaction conditions and chemical additions. In each test, 5–20 L/mins of N_2_ was added to 3.5 kg of dry sewage sludge. The effects of additions on bio-oil composition were studied by checking the reactions between the oil and catalyst (KOH, H_2_SO_4_, H_3_BO_3_, ZnCl_2_, and FeSO_4_). All catalysts enhanced calorific value, density, viscosity, and carbon content, while they reduced bio-oil production. Product quality was diminished by ZnCl_2_. KOH favored alkanes and monoaromatics, whereas H_2_SO_4_ and H_3_BO_3_ favored cyclics, ketones, alcohols, and nitriles but repressed amides and esters.

The MAP of rice straw was studied by Huang et al. [117]. The pyrolysis process was carried out in a single-mode microwave oven operating at a frequency of 2.45 GHz with a maximum power output of 2 kW. The pyrolysis efficiency was impacted by the particle size and the microwave exit power. Less microwave energy is needed for the pyrolysis of rice straw when the particles are tiny. Water and waste water treatment (metallic pollutant removal) showed promise in the solid fraction study (specific surface area, seta potential, and Cu_2_ adsorption). Half of the rice straw sample was converted into a rich flue gas consisting of 55 vol% H_2_, 17 vol% CO_2_, 13 vol% CO, and 10 vol% CH_4_. OPS biomass conversion was performed by Salema and Ani [118] using a microwave-assisted pyrolysis reactor with an overhead stirrer. Similar microwave apparatus and temperature measurements [108] were utilized in this study, but a three-necked glass cover was placed above the reactor in order to accommodate an overhead stirrer. In order to produce an inert environment, N_2_ was employed at a rate of 10 L/min for 5 min before the experiment and at a rate of 5 L/min during the operation. Both 300 W and 450 W exit outputs from the microwave were employed in these tests, with a reaction time of 25 min and 200 revolutions per minute (rpm). At 180 W and 450 W, the OPS did not pyrolyze without C_ac_. C_ac_ at 10% yielded the least bio-oil but the most bio-char. Bio-char production was greatest at 900 °C, whereas it was lowest at 400 °C and highest at 700 °C. Finally, mechanical stirrers have the potential to raise the temperature and heat transfer of microwave pyrolysis.

MAP for biomass (aspen hardwood pellets) treatment was studied by Moen et al. [119]. Catalysts such as chlorides, nitrates, metal oxides, and magnesium were used in a Sineo MAS-II batch microwave oven at 800 W continuous power. The production of liquid, pyrolysis gas, and heavy oil all rose in response to the addition of chlorides, nitrates, and metal oxides, but light oil production remained stable. Chlorides were used alone to boost bio-mass input liquid production from 35% to 41%. It was found that nitrates, metal oxides, and chlorides produced the highest percentages of gas (35.57%), char (32.01%), heavy oil (12.93%), and light oil (10.68%), respectively. Synthesis gas made up 69% of the pyrolysis gas volume, whereas CH_4_ only made up 18%. The MAP of maize stover (stalks, leaves, cobs, and husks) was performed by Yu et al. [120]. The input power ranged from 300 W to 900 W, and the response time was 60 min. This research examined the catalytic effects of NaOH and the MA properties of charcoal. Increases in gas and bio-oil production were accompanied by decreases in charcoal output when the microwave input power was raised from 300 W to 900 W. Without any additions, 900 W produced the most bio-oil (30.2%) and gas (46.7%), whereas 300 W created the most charcoal (69.3%). Increasing the power to 600 W raised the amount of bio-oil and gas produced to 29.5 and 45.7%, respectively, while decreasing the amount of charcoal produced.

Bio-oil was obtained from OPS using the MAP technology described by Salema and Ani [121]. In this study, char was used as an MA at weight ratios of 0:1, 0.25:1, 0.5:1, and 1:1 (biomass to char). The reaction was run at 450 W of microwave radiation for 25 min using N_2_ as the carrier gas at a flow rate of 20 L/min. The optimal ratio of biomass to MA was 1:0.5, which resulted in high bio-oil and gas production with little char formation. High-density polyethylene and aluminum/polymer laminates were studied by Palafox and Chase [122] using semi-batch MAP. They were studying the degradation of high-density polyethylene and aluminum/polymer laminates in a semi-batch bench-scale apparatus. Toothpaste tubing was used as an example of a laminated material to be treated with the novel process. Clean aluminum was recovered together with hydrocarbons, and the trial proved that the process has excellent potential for the treatment of plastic waste. MAP for the treatment of maize stover for bio-oil, syngas, and bio-char production was studied by Hanwu Lei et al. [123] by using particle size (0.5–4 mm), reaction time (4–22 min), and temperature (515–685 °C). A 1 L quartz flask was heated in a 700 W Sineo MAS II batch microwave oven. After 6 min of running at 50 and 160 °C per minute, the stage had heated to the proper level. The greatest volatile output was 76% (34% bio-oil and 42% biogas) during an 8 min reaction at 650 °C with 4 mm particles.

Using C_ac_, Fernandez et al. [124] compared conventional and microwave-assisted glycerol-to-syngas production. Gas output from glycerol conversion rose significantly with high syngas and H_2_/Co ratios after being heated in a microwave oven. MAP technology showed promising results in the breakdown process by decreasing the solid fraction yield. Syngas production was enhanced, and CO_2_ emissions were reduced by using carbonaceous catalysts. Activating Jatropha shell carbon was studied by Xin-hui et al. [125] by comparing microwave and traditional heating techniques. The microwave oven was a 3-kilowatt (2.45 GHz) model with a quartz pipe in its center. The experimental analysis was carried out using the statistical response surface (RSM) method. In this experiment, pyrolysis was performed using steam and CO_2_. The activation temperature, duration, and CO_2_ flow rate were all reduced by microwave heating, while the output yield was increased from 18.02% to 36.60%. Despite the carbon having the same porosity, this method reduced costs. The production of C_ac_ was unaffected by the use of a microwave-assisted heating method for steam activation, which quadrupled the pore volume and surface area. 

Table 2 summarizes the reviewed articles that use MAP technology on different types of biomass and obtain useful products, e.g., bio-oil, bio-char, etc. The research target and main findings of the review can be found in Table 2. It can be perceived from Table 2 that mostly MAP technology is used for treatment of agricultural crop residue or virgin biomass resources; however, a limited number of studies have tested sewage sludge as a biomass input for treatment. The research target, biomass input, product output, microwave power and frequency and findings of reviewed articles are listed in Table 2. One of the biggest difficulties in microwave pyrolysis is measuring temperature accurately since it impacts the conditions and effectiveness of the reaction. In order to minimize measurement oversights, selecting the right temperature sensor appears essential. The results from grounded thermocouple probes will not be as reliable since they sometimes interact with electromagnetic fields. In an effort to address this issue and improve the accuracy of temperature monitoring systems, grounded probe TCs may be used in conjunction with infrared optical pyrometers. Most microwave pyrolysis systems analyzed in the above-mentioned research, have on–off switching systems. However, having a temperature feedback control system is one of the crucial instruments for achieving a constant pyrolysis reaction state. Reaction conditions may be stabilized to create bio-products with decreased solidity and retention time. The viability of microwave pyrolysis has only been evaluated in laboratories. The viability of the system is determined by pyrolyzing only 3–400 g of biomass leftovers. Before commercialization, the technology has to be scaled up, but there has been no pilot testing. A continuous microwave pyrolysis system should be able to overcome many issues that other processes have, as pyrolysis is one of the finest ways to recover energy and chemicals from biomass residuals. Microwave pyrolysis cannot be used for all materials, e.g., transparent and dry materials, but it works efficiently in materials with high moisture content. In addition, transparent materials with added absorbers in the microwave assist in increasing overall temperature of the reaction. Another issue is the difficulty of monitoring temperature in a microwave environment, and thermal damage to treated materials is caused by the microwave’s non-uniform heating behavior. Because the homogeneity of the microwave field may be improved by enlarging the cavity, a specific instrument design is required. Additionally, microwave leakage poses a risk to human health, necessitating safety measures as well as cautious handling.

## 5. Conclusions and Future Work Suggestions

Microwave-assisted pyrolysis is a revolutionary approach for the effective in situ processing of biomass. Microwave heating is a practical option for recovering energy from biomass and turning it into usable goods. When compared to oils obtained through traditional pyrolysis, those obtained through this method yield fewer harmful substances and more chemicals of industrial importance. Microwave-assisted pyrolysis offers a promising opportunity to divert biomass from environmentally detrimental disposal techniques like landfilling and incineration while simultaneously providing a practical means of recovering marketable commodities from leftover ash. Parameters and operational variables that control this process have been the subject of some attention and may yet benefit from optimization. By defining the right process parameters (RT = residence time; HR = heating rate; Te = temperature; VR = vapor resentence time) through optimization, the microwave-assisted pyrolysis process may be taken to a larger scale.

The primary factor contributing to the price tag is the expense of the pyrolysis procedure. As a result, one way to lower product prices is to advance pyrolysis technology. The use of microwave technology in thermochemical processes like pyrolysis has been shown to save energy and money. Time and energy savings are only two of the many benefits of the microwave-assisted pyrolysis approach that cannot be achieved with more traditional heating methods. The type of feedstock utilized and the reaction time greatly influence the features of the pyrolysis process and the yield of the products it generates. The current study reviewed research articles that applied microwave-assisted pyrolysis techniques and yielded some useful products, e.g., bio-oil, bio-char, and charcoal, without negatively impacting the environment.

The use of MAP technology is mostly tested on a lab scale. There is currently limited pilot-scale testing using MAP technology for biomass conversion, which must be scaled up for commercialization. Also, various types of biomass belong to various categories that should be explored by using MAP technology. Future studies should pay attention to and work on these suggestions to investigate the performance of MAP both at the lab scale and on a large scale.

## Figures and Tables

**Table 1:** Process conditions and pyrolysis modes.

PT (Type)	RT (s)	HR (°C/s)	Te (°C)	VR (s)
**slow**	>290	0.10–10	400–900	≤550
**fast**	0.50 to 10	10–200	450–850	0.5–10
**flash**	<1	>1000	600–1200	<0.5

PT = pyrolysis mode; RT = residence time; HR = heating rate; Te = temperature; VR = vapor resentence time.

**Table 2:** Summary of reviewed articles to apply microwave pyrolysis for different biomass.

Studies	Research Target	Biomass Input	Product Output	Microwave Power	Microwave Frequency	Study Findings
Zhao et al. [95]	Investigated the effect of temperature on the MAP of biomass	wheat straw	fuels and chemicals	3000 W	2.45 GHz	It was found that the temperature has a significant impact on the performance of MAP.
Hu et al. [96]	Investigated the effect of microwave power level and catalysts on the products	solid residue (*Chlorella vulgaris*)	bio-oil	3750 W	2.45 GHz	Using C_ac_ as a catalyst yielded the best results for solid residue, and raising the microwave power level improved gas generation.
Ren et al. [97]	Examined the influence of reaction time and temperature	Douglas fir sawdust pellet	syngas, bio-oil, and charcoal	700 W	2450 MHz	It was discovered that increasing the reaction time and temperature increased the yield of volatiles.
Chen et al. [98]	Examined the catalytic effects of eight distinct inorganic additives	pine wood sawdust	Acetol formation	300–800 W	2.45 GHz	It was determined that each of the eight additives increased the output of the solid portion, whereas the yield of the gaseous and liquid products remained relatively unchanged.
Omar et al. [99]	Examined the characterization of oil palm empty fruit bunch (EFB)	Biomass (OP-EFB)	bio-oil	800 W	2450 MHz	Based on the results, EFB has tremendous potential as a raw material for microwave pyrolysis since its fuel and chemical qualities are on par with those of other biomass feedstock.
Hussain et al. [100]	Investigated the microwave–copper interaction of waste polystyrene and Makarwal coal co-pyrolysis at high temperature	waste polystyrene and Makarwal coal	acetylene and sulfur	3 kW	2450 MHz	The quantity of oil produced was observed to be greater than the amount of water.
Tian et al. [101].	The authors attempted to establish a relationship between the mass balance of the products and microwave exit power.	sewage sludge	bio-oil	400–600 W	2.45 GHz	At 400 W microwave exit power for 6 min, the highest bio-oil output (49.8%), highest calorific value (35.5 MJ/kg), and lowest density (929 kg/m^3^) were all achieved.
Du et al. [102]	Examined microwave exit power (160 W to 800 W), microwave heating time (up to 25 min), and catalyst dosage (0.0 to 0.5 catalyst/biomass ratio)	rice straw and sawdust	bio-oil	160–800 W	2.45 GHz	After 20 min of microwave irradiation (for both catalysts), it was found that the yields of bio-oil from rice straw stabilized.
Zeng et al. [103]	Evaluated the effect of MAP on the growth and morphology of biomass in the absence of a catalyst	methane	carbon nanostructures	700 W	2.45 GHz	The findings revealed that MAP is a novel and promising method for synthesizing carbon nanostructures.
Huang et al. [104]	Examined the effect of microwave heating on pyrolysis	rice straw	H_2_-rich fuel gas	3000 W	2.45 GHz	It was found that the hotspots generated by microwave ovens could be utilized for H_2_ production.
Fu et al. [105]	Investigated the effects of reaction conditions, such as the concentration and temperature of the reaction gas	methane	Syngas	0.7 KW	2450 MHz	It concluded that a nanowire of high purity could be synthesized using this novel method.
Andersson et al. [106]	Analyzed six distinct fractions of discarded electrical and electronic equipment	six different fractions of waste	Bio-oil and syngas	3 KW	2.45 GHz	This method was discovered to be effective at transforming wastes into more valuable substances.
Chemat and Poux [107]	Examined the effect of microwave heating on pyrolysis	urea	cyanuric acid	2 KW	2450 MHz	The reaction rates of heterogeneous pyrolysis were significantly accelerated.
Salema and Ani [108]	Examined the effect of microwave heating on pyrolysis	oil palm shell and fibers	bio-oil	1000 W	2.45 GHz	Large biomass particle sizes can be utilized in microwave reactors, resulting in cost reductions for grinding and moisture removal.
Lei et al. [109]	The effect of critical reaction conditions, including reaction time, temperature, and microwave exit power, was investigated and analyzed through response surface methodology (RSM).	dried grain	bio-oil	900 W	2450 MHz	The most effective reaction conditions for microwave pyrolysis of DDGS are reaction time and temperature.
Wan et al. [110]	Reactions to various catalysts were analyzed	dry corn stover and aspen wood pellets	bio-oil	875 W	2450 MHz	It was discovered that the catalysts may serve as an MA to accelerate the heating process.
Du et al. [111]	Examined the effect of microwave power on pyrolysis	Chlorella sp.	bio-oil compounds	1.25 kW	2.45 GHz	From 500 W to 1250 W, gas output increased by 12%, creating bio-oil molecules similar to fossil fuels including crude oil, gasoline, and diesel.
Lam et al. [36]	Examined the effectiveness of MAP on waste products	waste automotive engine oil	bio-oil compounds	5 kW	2450 MHz	MAP has great potential for recycling wastes like waste oils.
Bu et al. [112]	Microwave pyrolysis was used with C_ac_ as the catalyst.	lignocellulose biomass	Phenolic products	700 W	2450 MHz	The upgraded phenol may be utilized as fuel or as a feedstock for bio-based phenols.
Lam et al. [113]	Explored the microwave pyrolysis of automobile engine oil to learn about the properties of the incondensable gaseous produced	engine oil	hydrocarbon energy	(5 kW)	2450 MHz	Microwave irradiation with carbon bed aid strengthened the production of useful gases as an alternative source of hydrogen or hydrocarbon energy from waste oil.
Quan et al. [114]	Investigated biofuels with high concentrations of phenol and phenolics	biomass feedstock	phenol	700 W	2.45 GHz	C_ac_ was observed to substantially increase volatile yields.
Lam et al. [115]	Discussed the thermal cracking (pyrolysis) of used automobile engines	engine oils	metal contaminants	3 KW	2450 MHz	MAP is a promising method for treating residues such as engine lubricants.
Lin et al. [116]	This study investigated the impact of reaction parameters and chemical additives on the yield and quality of bio-oil produced.	sewage sludge	bio-oil	8.8 kW	2.45 GHz	Maximum bio-oil yield (30.4%) was obtained at a microwave exit power of 8.8 kW and a final pyrolysis temperature of 500 °C.
Huang et al. [117]	Examined the effect of microwave power on pyrolysis	rice straw	flue gas	2 kW	2.45 GHz	Lower microwave power would be suitable when the particle size is small.
Salema and Ani [118]	Explored the use of an overhead stirrer in a MAP reactor	Biomass (OPS)	bio-char	1000 W	2.45 GHz	Mechanical stirrers can achieve higher microwave pyrolysis temperatures, enhance heat transfer rate, remove hot spot phenomena, and reduce time and energy for complete pyrolysis. However, size and design must be maintained to prevent interference from electromagnetic waves.
Moen et al. [119]	Examined the effect of catalyst on pyrolysis	Aspen hardwood pellets	bio-oil	800 W	2.45 GHz	The maximum yields of gas (35.57%), char (32.01%), heavy oil (12.93%), and light oil (10.68%) were obtained with nitrates, without catalyst, metaloxides, and chlorides, respectively.
Yu et al. [120]	Examined the effect of microwave power on pyrolysis	corn stover	charcoal	900 W	2.45 GHz	Increasing the microwave input power from 300 W to 900 W increased the output of gas and bio-oil while decreasing the yield of charcoal.
Salema and Ani [121]	Examined the effect of MAP to produce quality products	OPS/OPF	bio-oil	1000 W	2.45 GHz	MAP can be used to convert waste materials such as OPS/OPF into bio-oil and other quality byproducts.
Palafox and Chase [122]	High-density polyethylene and aluminum/polymer laminates in a semi-batch-scale microwave reactor was evaluated.	polyethylene and aluminum/polymer laminates	Clean aluminum along with hydrocarbons	5 kW	2450 MHz	Toothpaste tubing was used as an example of a laminated material to be treated with the MAP. The experiment proved that the process has excellent potential for the treatment of plastic wastes such as toothpaste.
Hanwu Lei et al. [123]	Examined the effects of particle size (0.5–4 mm), reaction time (4–22 min), and temperature (515–615 °C) on microwave pyrolysis	corn stover	bio-oil	700 W	2450 MHz	Large biomass materials can undergo using MAP technology and generate useful products.
Fernandez at al. [124]	Examined the effect of MAP to produce quality products against a conventional heating method	glycerol	syngas	1 KW	2.45 GHz	The results demonstrated that microwave-assisted heating produced higher gas yields (converted from glycerol) with a high syngas content and a higher H_2_/Co ratio compared to conventional heating method.
Xin-hui et al. [125]	Steam and CO_2_ were used as activation agents for the pyrolysis process.	Jatropha hull	activated carbon	3 kW	2.45 GHz	Microwave heating increased the production yield (double) using CO_2_ as the activation agent. This made the process more economical, although the porosity of carbon was in the same order of magnitude.

## Data Availability

Data are available on reasonable request from the first author.
